# Supplementary material for: Deep Eutectic Solvents as a Green Tool for the Extraction of Bioactive Phenolic Compounds from Avocado Peels
Source: Molecules. 2022 Oct 6;27(19):6646. doi: 10.3390/molecules27196646 (PMC9572341; doi:10.3390/molecules27196646)
Supplement: Supplementary file 1 [file molecules-27-06646-s001.zip › molecules-1929866-supplementary.pdf]

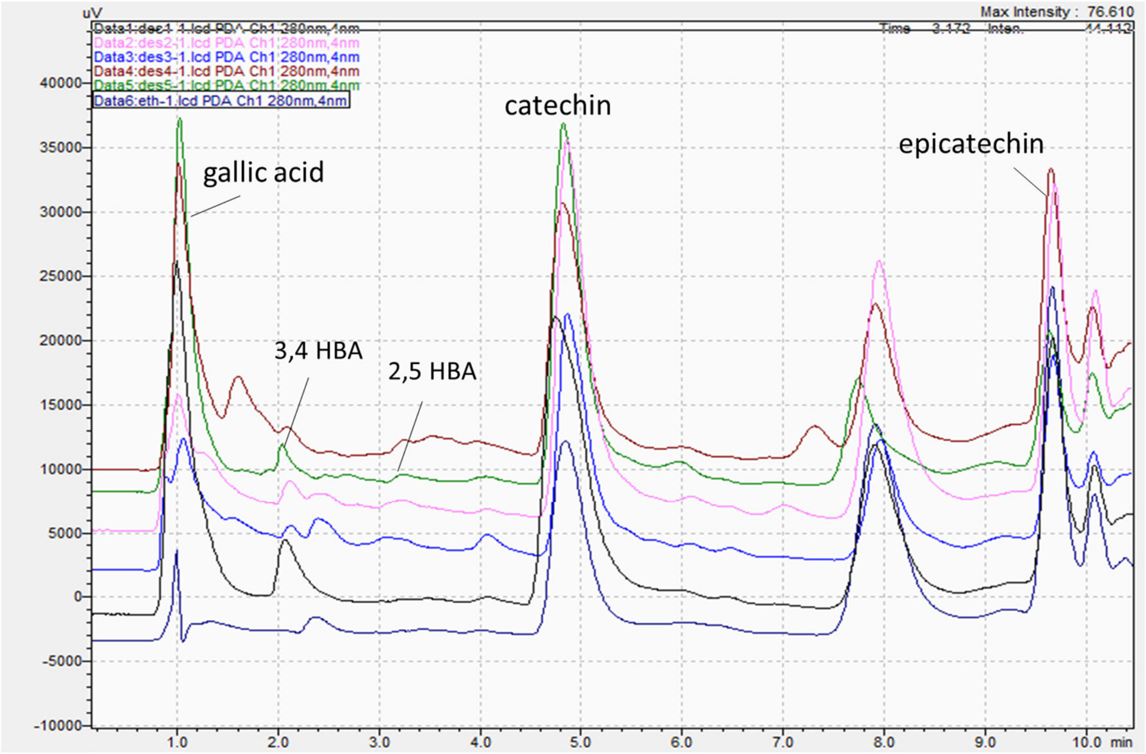

Figure S1. Example of a chromatogram of some identified compounds at 280 nm from the studied avocado peel extracts.

Table S1. Pearson's correlation coefficients for the total phenolic content (TPC), total flavonoid content (TFC) and antioxidant capacity (FRAP and AAT) of different extracts from avocado peel. Significant correlations are marked in bold.

	TPC	TFC	FRAP	AAT
		Ethanol 96%		
**TPC**	1.00	-	-	-
**TFC**	**0.61**	1.00	-	-
**FRAP**	**0.99**	**0.71**	1.00	-
**AAT**	**-0.49**	**0.39**	-0.37	1.00
		DES 1		
**TPC**	1.00	-	-	-
**TFC**	**-0.17**	1.00	-	-
**FRAP**	-1.00	-1.00	1.00	-
**AAT**	**1.00**	-0.18	-1.00	1.00
		DES 2		
**TPC**	1.00	-	-	-
**TFC**	**0.54**	1.00	-	-
**FRAP**	-0.12	**-0.90**	1.00	-
**AAT**	**0.79**	**0.94**	-0.71	1.00
		DES 3		
**TPC**	1.00	-	-	-
**TFC**	**1.00**	1.00	-	-
**FRAP**	-0.78	-1.00	1.00	-
**AAT**	**1.00**	**1.00**	1.00	1.00
		DES 4		
**TPC**	1.00	-	-	-
**TFC**	-0.96	1.00	-	-
**FRAP**	0.98	**-0.88**	1.00	-

**AAT**	1.00	-1.00	**1.00**	1.00
		DES 5		
**TPC**	1.00	-	-	-
**TFC**	**1.00**	1.00	-	-
**FRAP**	-1.00	-1.00	1.00	-
**AAT**	-1.00	**1.00**	-0.69	1.00
